# Characterization of immortalized human islet stromal cells reveals a MSC-like profile with pancreatic features

**DOI:** 10.1186/s13287-020-01649-z

**Published:** 2020-04-17

**Authors:** Orianne Villard, Mathieu Armanet, Guilhem Couderc, Claire Bony, Jerome Moreaux, Daniele Noël, John DeVos, Bernard Klein, Jean-Luc Veyrune, Anne Wojtusciszyn

**Affiliations:** 1 Laboratory of Cell Therapy for Diabetes, Institute of Regenerative Medicine and Biotherapy, Univ. Montpellier, CHU Montpellier, Montpellier, France; 2 Department of Endocrinology, Diabetes, and Nutrition, Univ. Montpellier, CHU Montpellier, Montpellier, France; 3 Cell Therapy Unit, Hospital Saint- Louis, AP-HP, Paris, France; 4 Department of Endocrinology, Diabetology and Metabolism, Lausanne University Hospital, 8 avenue de la Sallaz – 1011, Lausanne, Switzerland; 5 Department of Biological Haematology, Univ. Montpellier, CHU Montpellier, Montpellier, France; 6 Department of Cell and Tissue Engineering, Univ. Montpellier, CHU Montpellier, Montpellier, France; 7 IRMB, INSERM U 1183, Univ Montpellier, INSERM, Montpellier, France; 8 IGH, Univ Montpellier, CNRS, Montpellier, France

**Keywords:** Pancreatic islets, Mesenchymal stromal cells, Conditioned medium, Extracellular matrix, Cell therapy

## Abstract

**Background:**

Mesenchymal stromal cells (MSCs) represent an interesting tool to improve pancreatic islet transplantation. They have immunomodulatory properties and secrete supportive proteins. However, the functional properties of MSCs vary according to many factors such as donor characteristics, tissue origin, or isolation methods. To counteract this heterogeneity, we aimed to immortalize and characterize adherent cells derived from human pancreatic islets (hISCs), using phenotypic, transcriptomic, and functional analysis.

**Methods:**

Adherent cells derived from human islets in culture were infected with a hTERT retrovirus vector and then characterized by microarray hybridization, flow cytometry analysis, and immunofluorescence assays. Osteogenic, adipogenic, and chondrogenic differentiation as well as PBMC proliferation suppression assays were used to compare the functional abilities of hISCs and MSCs. Extracellular matrix (ECM) gene expression profile analysis was performed using the SAM (Significance Analysis of Microarrays) software, and protein expression was confirmed by western blotting.

**Results:**

hISCs kept an unlimited proliferative potential. They exhibited several properties of MSCs such as CD73, CD90, and CD105 expression and differentiation capacity. From a functional point of view, hISCs inhibited the proliferation of activated peripheral blood mononuclear cells. The transcriptomic profile of hISCs highly clusterized with bone marrow (BM)-MSCs and revealed a differential enrichment of genes involved in the organization of the ECM. Indeed, the expression and secretion profiles of ECM proteins including collagens I, IV, and VI, fibronectin, and laminins, known to be expressed in abundance around and within the islets, were different between hISCs and BM-MSCs.

**Conclusion:**

We generated a new human cell line from pancreatic islets, with MSCs properties and retaining some pancreatic specificities related to the production of ECM proteins. hISCs appear as a very promising tool in islet transplantation by their availability (as a source of inexhaustible source of cells) and ability to secrete a supportive “pancreatic” microenvironment.

## Introduction

Mesenchymal stem/stromal cells (MSCs) are considered as a promising approach for regenerative medicine and cellular therapy applications. Their characterization meets the following criteria: the capacity of adhesion to plastic, the expression of surface antigen markers including CD73, CD90, and CD105, and the ability to differentiate into osteocytes, adipocytes, and chondrocytes [1]. Thanks to their immunomodulatory properties and their ability to secrete trophic factors, they are expected to revolution the fate of transplantation and cellular therapy.

In diabetes cell therapy applications, such as islet transplantation, the use of MSCs could be an interesting field of development to improve the quality of grafted islet [2]. Indeed, during islet isolation and culture prior to transplantation, islets are weakened leading to a significant loss of functional β cell mass [3]. This phenomenon is accentuated in the few days following infusion into the liver, due to coagulation, inflammatory, and hypoxic reactions [4], and islet graft remains a marginal mass transplantation [5]*.* To overcome these adverse events, MSCs could address the therapeutic challenge of preserving the β cell mass and function after isolation and transplantation.

Co-culture or co-transplantation of isolated islets with MSCs confirmed beneficial effects on β cell function and survival as well as on islet engraftment. Modulation of the host niche by the presence of MSCs promotes capillary formation and facilitates islet revascularization by the secretion of vascular endothelial growth factor [6, 7]. Hepatocyte growth factor (HGF) and metalloproteinases (MMPs) 2 and 9 released by human MSCs prolong grafted islet survival by decreasing activation of T cells [8]. Both MMPs and HGF also seem to protect islets from pro-inflammatory cytokines, in vitro [9]. More recently, it was suggested that extracellular matrix (ECM) proteins present in conditioned media of MSCs derived from human adipose tissue were beneficial for β cell function [10]. Finally, all these studies emphasize the importance of the protective effects of the soluble factors secreted by MSCs [11, 12]. This raises the possibility of using a cell-free approach to improve clinical islet graft outcomes [13]. However, these in vivo and in vitro results have not yet been confirmed in human clinical application.

Bone marrow (BM)-MSCs and adipose tissue-derived stem cells (ASCs) are the sources of MSC primarily used for experimental and clinical applications. Although both are easily available, several obstacles limit their use in routine. First, reproducibility of primary MSC effects is limited by intra- and inter-individual heterogeneity [14]. MSCs are found at a low frequency in other tissues and require an extensive in vitro expansion following isolation. This step of cellular amplification, even for BM-MSCs or ASCs, can delay their use in the emergency context of transplantation [15]. Moreover, they display finite life spans due to replicative senescence of MSCs in culture [16]. Finally, functional properties of MSCs differ according to their tissue origin with differences at the phenotypic, transcriptomic, and proteomic levels [17]. Thus, the question of the best source of human MSCs as supportive cells to improve human islet graft quality has recently emerged [18].

The use of MSCs originating from the pancreas appears to be a better option in the context of diabetes cell therapy. In a murine model, the pancreatic mesenchyme was recognized to positively regulate the final number of β cells generated from embryonic pancreas [19]. In addition, the species origin of supportive microenvironment is also crucial; human β cell function was improved with human-derived ECM proteins as compared to non-human proteins [20].

Accumulating evidence suggested the presence of proliferative cells with a mesenchymal phenotype after several days of culture of extremely pure adult human islets [21, 22]. Having an immortalized source of MSCs from human pancreas would be of great interest for a potential application in the context of islet transplantation. In the present study, we first aimed to immortalize adherent and proliferative cells derived from human pancreatic islets and then to characterize and compare them with human BM-MSCs using phenotypic, transcriptomic, and functional analysis.

## Materials and methods

### Isolation, immortalization, and culture of human islet-derived stromal cells (hISCs)

Human pancreases were obtained from brain-dead non-diabetic donors with prior consent for research use (after informed consent from the donor’s family) in agreement with the French regulation “Agence de la Biomédecine” (registration number: PFS13-006 and PFS13-008) and the “Ministère de l’Enseignement supérieur et de la Recherche” (registration number: DC no. 2014-2473 and 2016-2716/AC: 2017-3039). Islets were isolated by collagenase digestion followed by density gradient purification. After purification, dithizone-stained islets were carefully handpicked and seeded into 6-well plates. These selected islets were cultured in RPMI-1640 medium supplemented with 10% fetal bovine serum, 10 mM HEPES, 1 mM sodium pyruvate, 2 mM glutamine, 100 IU/ml penicillin and 100 μg/ml streptomycin (Life Technologies, Courtaboeuf, France), and ß-mercaptoethanol 71.5 μM (Merck, Fontenay-sur-bois, France) (hereafter defined as basal medium) and maintained in a humidified incubator at 37 °C and 5% CO_2_. After 3–5 days, the medium was replaced by fresh medium previously described and supplemented with 10 ng/ml fibroblast growth factor 2 (FGF2) and 10 ng/ml epidermal growth factor (EGF) (R&D Systems, Minneapolis, USA), hereafter referred as human islet-derived stromal cell (hISC) complete medium.

After 3 weeks of culture, human telomerase reverse transcriptase (hTERT) retroviral vector infection was performed. The full-length hTERT cDNA in pBabeHygrohTERT vector was provided by Dr. Weinberg [23]. This construct was first cut with Sal I. The restriction site has been blunted with klenow (New England Biolabs, Evry, France) and then cut with EcoR I. This fragment containing the full-length cDNA for hTERT was inserted in pEGN-MCS vector [24] at EcoR I and BamH I. This vector contains a green fluorescent protein (GFP) marker in fusion with the neo gene. Thus, cells integrating this vector express GFP continuously and survive after neomycin treatment. The construct and the empty retroviral vector pEGN-MCS were transfected into the HEK293E17 packaging cell line (Transgene). Supernatants derived from transfected HEK293E17 packaging cell line were co-cultivated with islet cells in the presence of polybrene (10 μg/mL, Sigma-Aldrich, Saint Louis, MO, USA) for 4 h. The cells were then washed and incubated in fresh hISC complete medium. Two additional rounds of infection were performed in the next 2 days. Immortalized cells were first selected after addition of G418, an analog of neomycin (0.7 mg/l, Life Technologies) in culture medium and then by cell sorting on a FACSCalibur flow cytometer (Becton Dickinson Bioscience) for GFP expression. Selected GFP-positive cells were defined as hISCs.

### Flow cytometry analysis

For immunophenotyping hISC were trypsinized and resuspended in PBS containing 0.1% BSA and 2 mM EDTA. Then, cells were incubated for 30 min at 4 °C with monoclonal antibodies against CD13, CD73, CD90, CD105, CD11b, CD14, CD31, CD34, CD45, and HLA-DR conjugated with either allophycocyanin (APC), phycoerythrin (PE), BV421, BV605, Alexa 647, peridinin chloryophyll-protein complex (PerCP), V500, or matched isotype control (BD Biosciences). Data were acquired and analyzed through a multiparameter Gallio flow cytometer with the Kaluza software (Beckman Coulter).

### Immunofluorescence analysis

Expressions of vimentin, synaptophysin, and chromogranin A were analyzed by immunofluorescence. Briefly, hISCs and human BM-MSCs were seeded on coverslips for 24 h at 37 °C/5% CO_2_. These coverslips were rinsed with PBS solution, fixed in 4% paraformaldehyde solution (PFA) for 15 min, rinsed again with PBS solution, and treated for 15 min with 0.1% Triton X-100. Nonspecific binding sites were blocked with 0.1% BSA solution for 60 min. Cells were then incubated overnight with primary antibodies in blocking solution: anti-vimentin (V9, Ventana Roche Diagnostic, Meylan, France), anti-synaptophysin (dilution 1:100, Novocastra Leica, Nanterre, France), and anti-chromogranin A (LK2H10, Ventana). Cells were then washed and incubated with specific secondary antibodies coupled to Alexa 555 (Jackson ImmunoResearch, Cambridgeshire, UK). The cell nuclei were stained with 2.5 μg/mL DAPI. Specificity of the different immunostainings was confirmed with cells in which primary antibodies were omitted. As positive control for anti-chromogranin A and anti-synaptophysin antibodies, human dispersed islets were used as previously described [25]. Cells were examined with a fluorescence microscope equipped with an Axiocam camera (Zeiss, Germany).

### In vitro osteogenic, adipogenic, and chondrogenic differentiation assay

Cell differentiation into osteogenic, adipogenic, and chondrogenic lineages was performed on hISCs, after 12 to 20 passages, using well-established protocols [17]. Human BM-MSCs, at passage 4 or earlier, were used as positive control. For osteogenic differentiation, hISCs were plated at a density of 3 × 10^3^ cells/cm^2^ in hISC complete medium. At confluence (between days 5 and 7), the medium was replaced by the osteogenic differentiation medium consisting in DMEM supplemented with 10% FBS, 2 mM glutamine (Lonza), 100 U/mL penicillin, 100 μg/mL streptomycin, 50 μg/mL ascorbic acid, 100 nM dexamethasone, and 3 mM NaH_2_PO4 (all from Sigma-Aldrich). The medium was changed every 3 days for 21 days. Cells were then rinsed with PBS, fixed with 95% ethanol for 30 min, and incubated with Alizarin Red solution for 5 min. Accumulation of calcium deposit matrix was observed by optic microscopy.

For adipogenic differentiation, hISCs were plated at a density of 8 × 10^3^ cells/cm^2^ in hISC medium. At confluence (between days 3 and 5), the medium was replaced by the adipogenic differentiation medium consisting in DMEM/F12 supplemented with 5% FBS, 100 U/mL penicillin, 100 μg/mL streptomycin, 16 μM biotin, 18 μM pantothenic acid, 100 μM ascorbic acid, 60 μM indomethacin, 450 μM IBMX, 1 μM dexamethasone, and 3 μM rosiglitazone (all from Sigma-Aldrich). The medium was changed every 3 days for 21 days. Cells were then fixed with 3% glutaraldehyde for 1 h and rinsed with 60% isopropanol. Then, cells were stained with Oil Red O dye (Sigma-Aldrich) for 2 h. Lipid droplet inclusions within the cells were observed by microscopy. Chondrogenic differentiation was induced by pelleting 2.5 × 10^5^ cells in 500 μL of DMEM medium supplemented with 10 ng/mL transforming growth factor (TGF)-β3 (R&D Systems), 100 U/mL penicillin, 100 μg/mL streptomycin, 1 mM sodium pyruvate, 170 μM ascorbic-2-phosphate acid, 350 μM proline, and 1% insulin–transferrin–selenic acid media supplement (Lonza). Media were changed every 2 days for 21 days. The pellets were then fixed, embedded in paraffin, and sectioned for histological staining with 0.1% Safranin O.

### Real-time quantitative polymerase chain reaction

Following differentiation into adipogenic lineage, total RNA was extracted using RNeasy mini kit and Qiacube robotic workstation (Qiagen, Courtaboeuf, France). RNA quality and concentration were determined on a NanoDrop spectrophotometer (Thermo Scientific, Waltham, USA). cDNA was synthesized using the Moloney Murine Leukemia Virus Reverse Transcriptase (Life Technologies) according to the manufacturer’s protocol. Quantitative real-time PCR was performed using the ViiA™7 Real-Time PCR System (Life Technologies) with SYBR green I Master-mix kit (Roche Diagnostics). Specific oligonucleotide primers were as follows: 5′-CCAGAAAGCGATTCCTTCAC-3′ + 5′-TGCAACCACTGGATCTGTTC-3′ for peroxisome proliferator-activated receptor gamma (PPARγ), 5′-ATGGGATGGAAAATCAACCA-3′ + 5′-GTGGAAGTGACGCCTTTCAT-3′ for fatty acid-binding protein 4 (FABP4), and 5′-GTCCGTGGCTACCTGTCATT-3′ + 5′-TGGATCGAGGCCAGTAATTC-3′ for lipoprotein lipase (LPL). The housekeeping gene (control) was ribosomal protein S9 (RPS9) (5′-GATTACATCCTGGGCCTGAA-3′ + 5′-ATGAAGGACGGGATGTTCAC-3′). Results were provided as relative expression to the housekeeping gene using the formula 2^−∆CT^.

### PBMC proliferation suppression assay

Peripheral blood mononuclear cells (PBMCs) were labeled with CellTrace Violet (CTV, 5 μM) (Life Technologies) to track proliferation and activated with 2.5 μg/mL phytohemagglutinin (PHA) (Sigma). hISCs or BM-MSCs were seeded in 96 well-plates at four densities (5 × 10^3^, 10^4^, 2 × 10^4^, or 4 × 10^4^ cells/well) in respective media during 24 h to allow cells to adhere. Then, activated PBMCs were added at the concentration of 2.10^5^ cells/well on hISCs or BM-MSCs in co-culture medium consisting in IMDM, 25 mM HEPES, supplemented with 10% inactivated fetal bovine serum, 1 mM sodium pyruvate, 0.1 mM non-essential amino-acids, 0.25 mM beta-mercaptoethanol, 2 mM glutamine, 100 IU/mL penicillin, and 100 μg/mL streptomycin. As control condition, activated PBMCs were cultured alone. After 96 h of co-culture, PBMCs were harvested and proliferation was quantified by flow cytometry using a FACSCanto cytometer (BD Bioscience) on the basis of CTV dilution. Results are expressed as the percentage of proliferation of stimulated PBMCs and normalized to 100% for activated PBMCs alone.

### Microarray hybridization and gene expression profiling analysis

RNA from hISCs (*n* = 5) and human pancreatic islets (*n* = 1) was extracted using the RNeasy kit (Qiagen). cRNA preparation and hybridization to the HG-U133 2.0 plus microarray (Affymetrix, Santa Clara, USA) were done as previously described [26]. Public microarray datasets were obtained with Gene Expression Omnibus (GEO, http:// www.ncbi.nlm.nih.gov/geo/) analysis from NCBI and selected based on the same platform (Affymetrix HG-U133 2.0 plus). Gene expression data of human BM-MSCs (GSE 9894 [27] GSE6460 [28]), human ASCs (GSE61302) expanded pancreatic islet cells, and human pancreatic islets (GSE15543 [22]) were downloaded from GEO. Normalization was completed using GC-RMA. Gene expression data were analyzed using the SAM (Significance Analysis of Microarrays) software [29] as published [30]. All these analyses have been done with R.2.10.1 (http://www.r-project.org/), bioconductor version 2.5, and Genomicscape (http://www.genomicscape.com) [31]. Genes differentially expressed between cell populations were identified using the SAM package on R software (fold change ≥ 2, false discovery rate (FDR) ≤ 0.05, 1000 permutations). Significantly enriched pathways were identified using Reactome Functional Interaction Cytoscape plugin (http://www.cytoscape.org/). Clustering was performed and visualized with Cluster and TreeView [32]. Mesenchymal stem cell and endocrine gene lists were generated using published transcriptomic data [22] and literature data.

### Extracellular matrix secretion

To determine whether ECM proteins were secreted by hISCs, 90% confluent cells were incubated in 10 mL of serum-free hISC complete medium. The conditioned medium was harvested after 48 h and cleared from floating cells and debris by centrifugation. These conditioned media and hISC lysates were assayed for collagens I, IV, and VI, fibronectin, and laminin by western blotting.

### Western blotting

Samples were fractioned by electrophoresis in 4–12% polyacrylamide gels (Thermo Scientific). Proteins were transferred electrically onto polyvinylidene fluoride (PVDF) membranes (Millipore) using a constant current of 400 mA for 80 min. PVDF membranes were saturated with 5% nonfat dried milk and 0.1% Tween-20 for 1 h and then probed with the following primary antibodies overnight at 4 °C: mouse anti-type I collagen (dilution 1:200, Santa Cruz), rabbit anti-type IV collagen (dilution 1:400, AbD Serotec), rabbit anti-type VI collagen (dilution 1:2000, Abcam), rabbit anti-fibronectin (dilution 1:1000, Santa Cruz), rabbit anti-laminin (dilution 1:500, Sigma Aldrich), and mouse anti-β-actin (dilution 1:10000, Sigma Aldrich). After 60 min of incubation at room temperature with appropriate horseradish peroxidase-conjugated secondary antibodies (diluted 1:2000, Bio-Rad Laboratories), proteins were visualized by enhanced chemiluminescence on ChemiDoc camera (Bio-Rad).

### Statistical analysis

Data are presented as mean ± SEM. Statistical analyses were carried out by Student *t* test using GraphPad Prism 8.1.2 (GraphPad Software, San Diego, CA, USA). A *p* value ≤ 0.05 was considered to be statistically significant.

## Results

### Immortalization and proliferation of human islet-derived cells

**Fig. 1:**
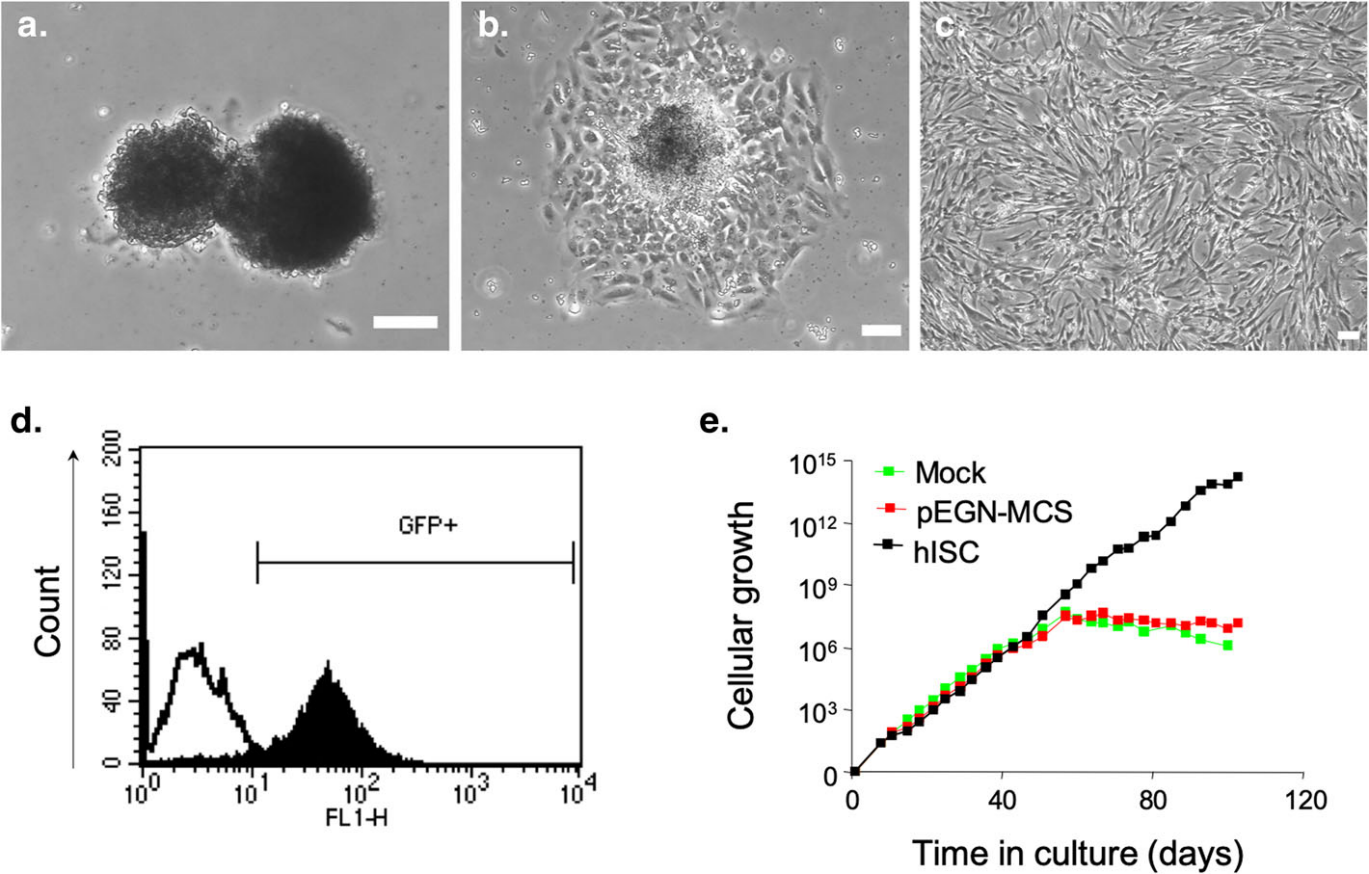
Isolation and selection of immortalized islet-derived cells. Phase-contrast microscopic view of pancreatic islets handpicked after dithizone staining (**a**, day 1). Islets adhered and cells spread-out (**b**, day 3) until becoming a monocellular layer of fibroblast-like cells (**c**, day 40). Scale bar = 100 μm. Efficiency of hTERT retroviral infection was assessed by neomycin resistance and GFP expression. GFP-positive cells were individualized by flow cytometry (**d**). GFP-sorted cells, defined as hISCs, did not shown senescence (black line) while non-infected cells (Mock, green line) or empty vector-transduced cells (pEGN-MCS, red line) stopped proliferating after 50 days (**e**)

Following dithizone staining, highly pure islets were selected by handpicking and cultured in hISC basal medium (Fig. 1a). After 3–5 days of culture, the basal medium was replaced by hISC complete medium. Herein, islets adhered with cells spreading out over the culture plate, around the islets (Fig. 1b). Progressively spreading cells extended radially and formed a monolayer of cells exhibiting a fibroblast-like shape, after 3 weeks (Fig. 1c). At this time, the medium was replaced and hTERT retroviral vector infection was performed. After 48 h of neomycin treatment, expression of the construct was assessed by flow cytometry using GFP as a marker. One hundred percent of the neomycin resistant cells expressed GFP (Fig. 1d) and were thereafter defined as hISCs. Although GFP detection by flow cytometry constituted a good evidence for vector insertion, we confirmed the expression of hTERT by RT-PCR. The amount of hTERT mRNA in hISCs was 6000-fold higher than hematopoietic stem cell line known to express hTERT (data not shown). During the first 40 days, hTERT-transduced cells as well as pEGN-MCS-transduced or Mock cells proliferated with a population doubling rate of about 3 days. However, after 50 days, non-hTERT-transduced cells gradually stopped proliferating and died at about 80 days (about 20 doubling times) whereas hTERT-transduced cells continued to proliferate (Fig. 1e).

### hISCs express a mesenchymal phenotype

**Fig. 2:**
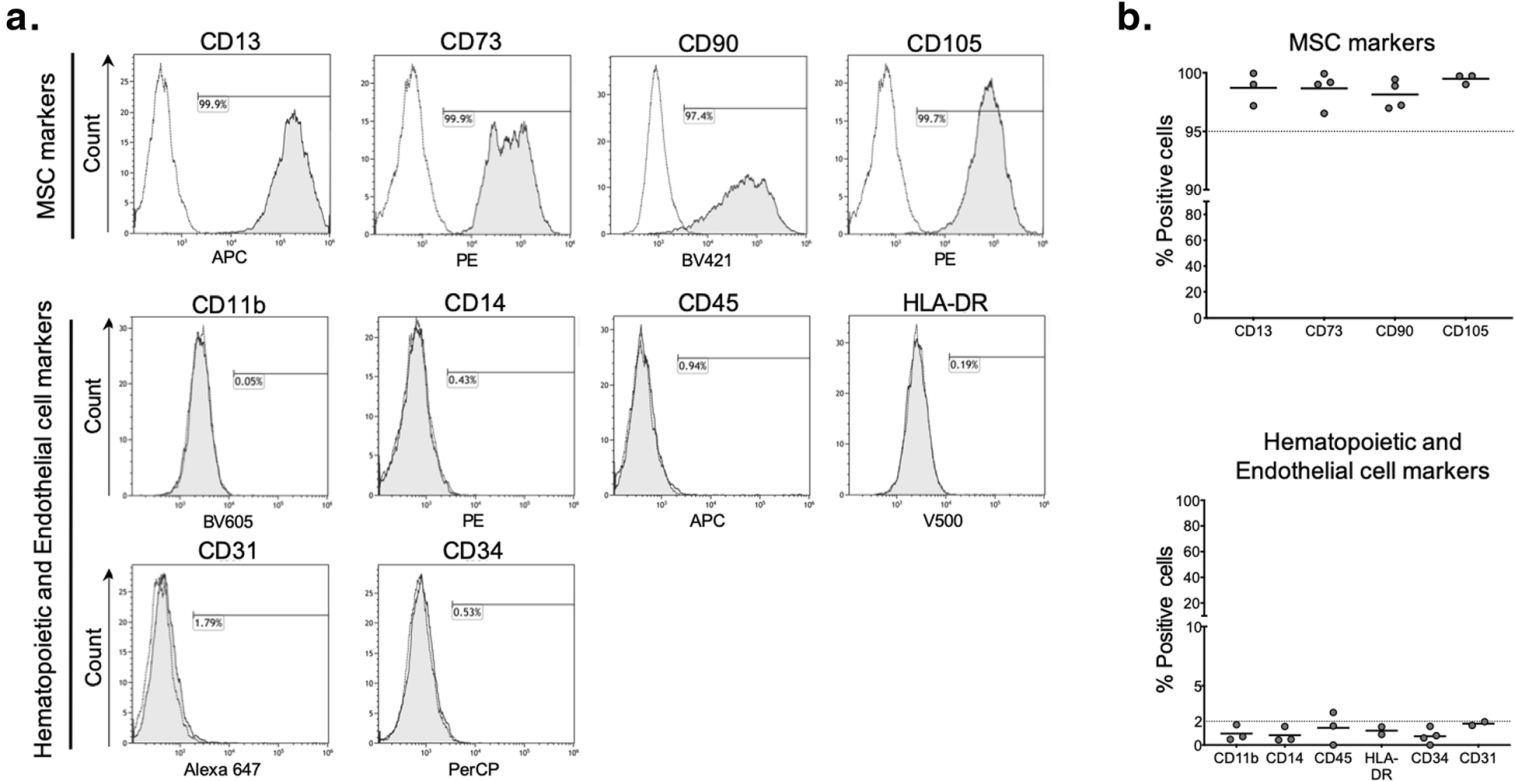
hISCs express MSC cell surface markers. Representative flow cytometric analysis of hISCs for specific MSC markers CD13, CD73, CD90, and CD105 and hematopoietic and endothelial markers CD11b, CD14, CD45, HLA-DR, CD31, and CD34 (**a**). More than 95% of hISCs were positive for MSC markers, and less than 2.5% of hISCs were positive for hematopoietic and endothelial markers. Results from 2 to 4 different experiments are shown (**b**)

**Fig. 3:**
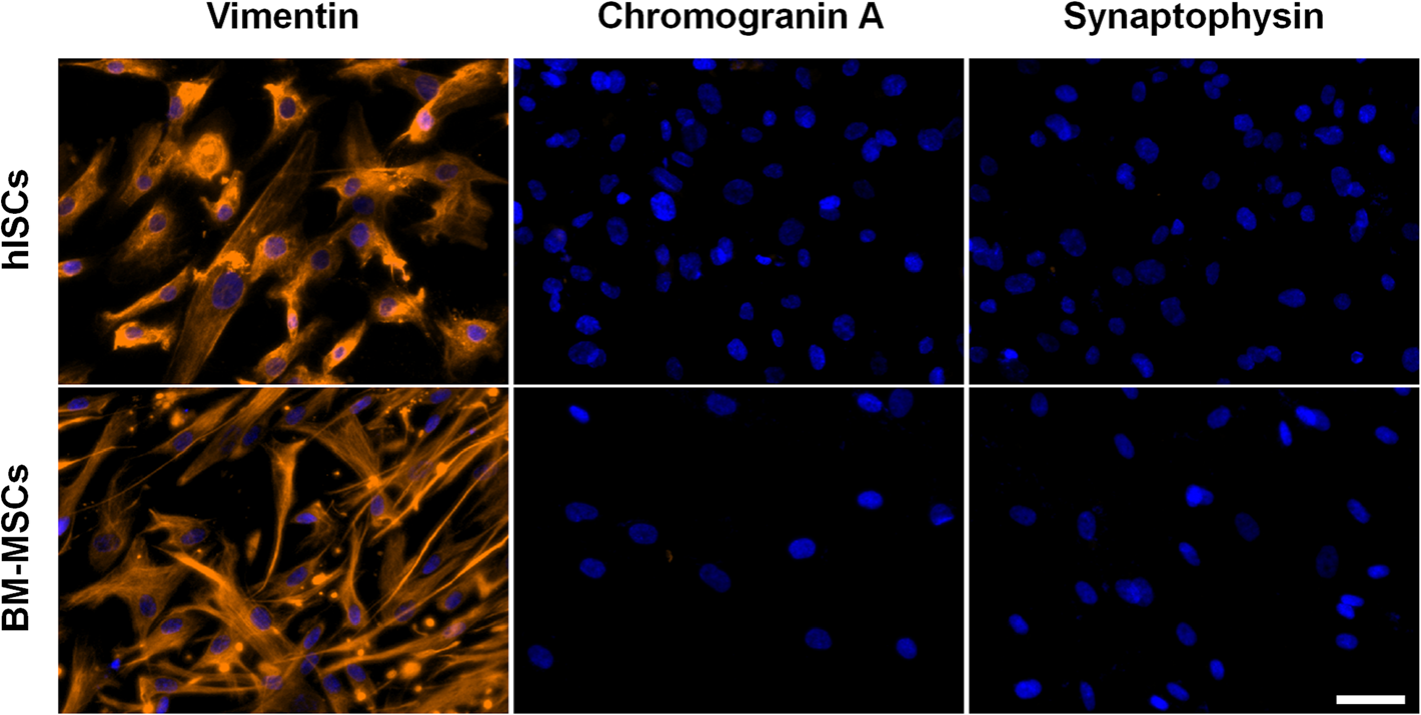
hISCs express vimentin and were negative for chromogranin A and synaptophysin. Immunofluorescence staining showed that both hISCs and BM-MSCs were positive for vimentin (orange) whereas they were negative for chromogranin A and synaptophysin. Nuclei were stained using DAPI (blue). Results are representative of three independent experiments. Scale bar = 50 μm

As hISCs exhibited a fibroblast-like shape with high adhesive properties, we went further in their characterization analyzing the expression of specific MSC surface antigens by FACS analysis. hISCs strongly expressed CD13, CD73, CD90, and CD105 (> 95%) and were negative for hematopoietic and endothelial markers such as CD11b, CD14, CD45, HLA-DR, CD31, and CD34 (Fig. 2). Moreover, immunofluorescence analysis revealed a positive cytoplasmic staining for vimentin in hISCs and BM-MSCs whereas both cell types were negative for the endocrine markers synaptophysin and chromogranin A (Fig. 3).

### Osteogenic and adipogenic differentiation of hISCs

**Fig. 4:**
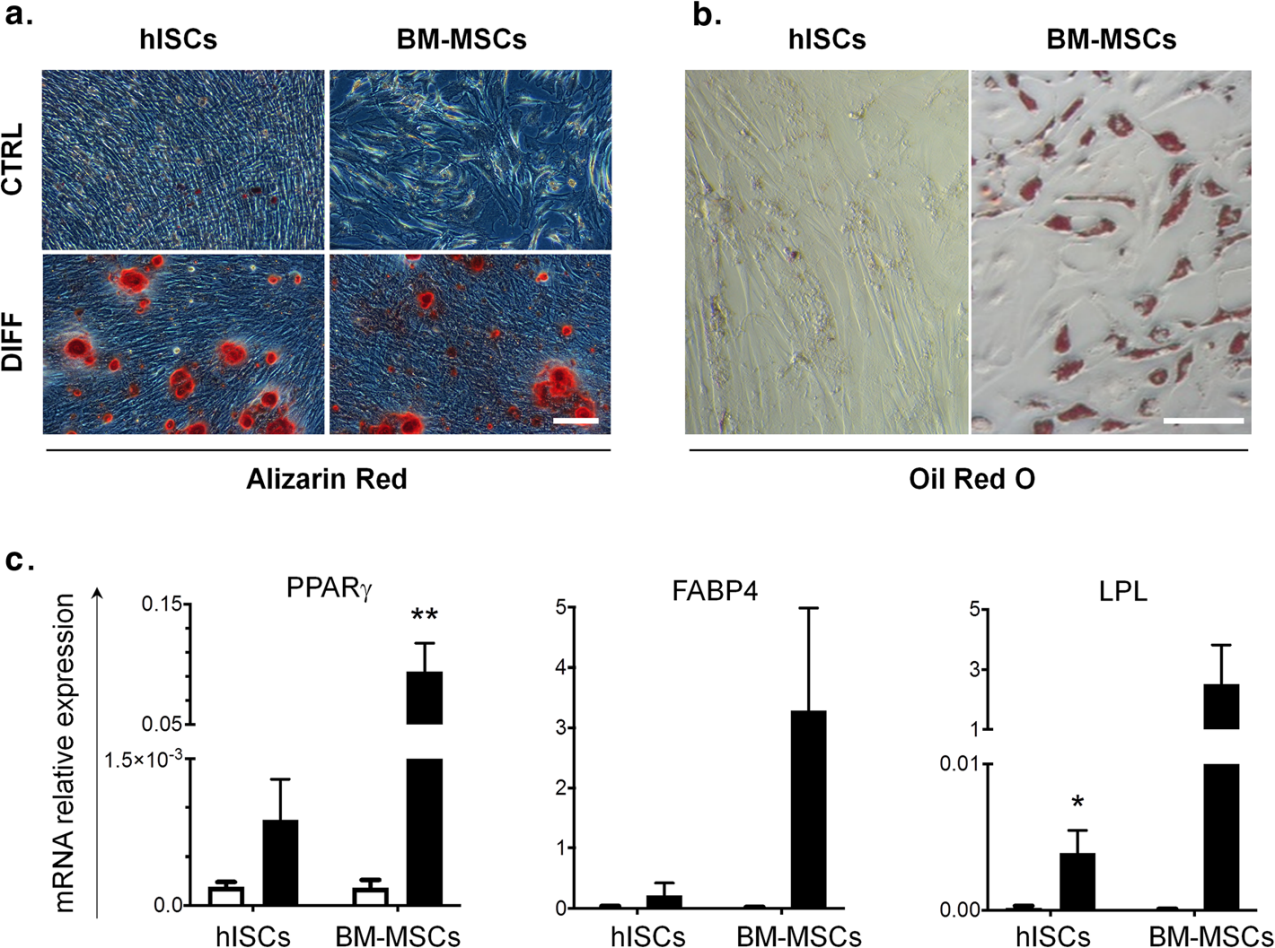
hISCs differentiate into osteogenic lineage and initiate adipogenic differentiation under inductive conditions. Phase-contrast microscopic view of hISCs and BM-MSCs cultured either in osteogenic differentiation medium (DIFF) (**a**) or in adipogenic differentiation medium (**b**). The deposition of calcified and mineralized ECM and the inclusion of lipid droplets were visualized by Alizarin Red and Oil Red O staining, respectively. Scale bar = 100 μm. mRNA expression of adipogenic genes (PPARγ, FABP4, and LPL) in hISCs and BM-MSCs was increased after 21 days in adipogenic differentiation medium (black column) as compared to day 0 (white column) (**c**). Values are normalized to RPS9 expression; *n* = 4. *p < 0.05, **p < 0.01 in differentiated versus undifferentiated cells

To assess their mesenchymal profile, hISCs were stimulated towards osteogenesis and adipogenesis. Both hISCs and BM-MSCs were able to differentiate into osteoblasts as assessed by Alizarin Red S staining showing red calcium deposits in sections (Fig. 4a). After adipogenic induction, hISCs changed their morphology towards a rounder shape but no lipidic inclusions were observed after Oil Red O staining as compared to the positive control BM-MSCs (Fig. 4b). However, the differentiation protocol increased at least the mRNA level of genes encoding for the adipocytic markers lipoprotein lipase (LPL), peroxisome proliferator-activated receptor gamma (PPARγ), and fatty acid-binding protein 4 (FABP4), in hISCs (Fig. 4c). Chondrogenic differentiation was also tested in a three-dimensional micropellet culture system with TGF-β3 enriched chondroinductive medium. However, the absence of chondrocyte gene upregulation (type II collagen, Sox9, and aggrecan) and positive Safranin O staining indicated no chondrogenic differentiation (data not shown).

### hISCs inhibit PBMC proliferation

**Fig. 5:**
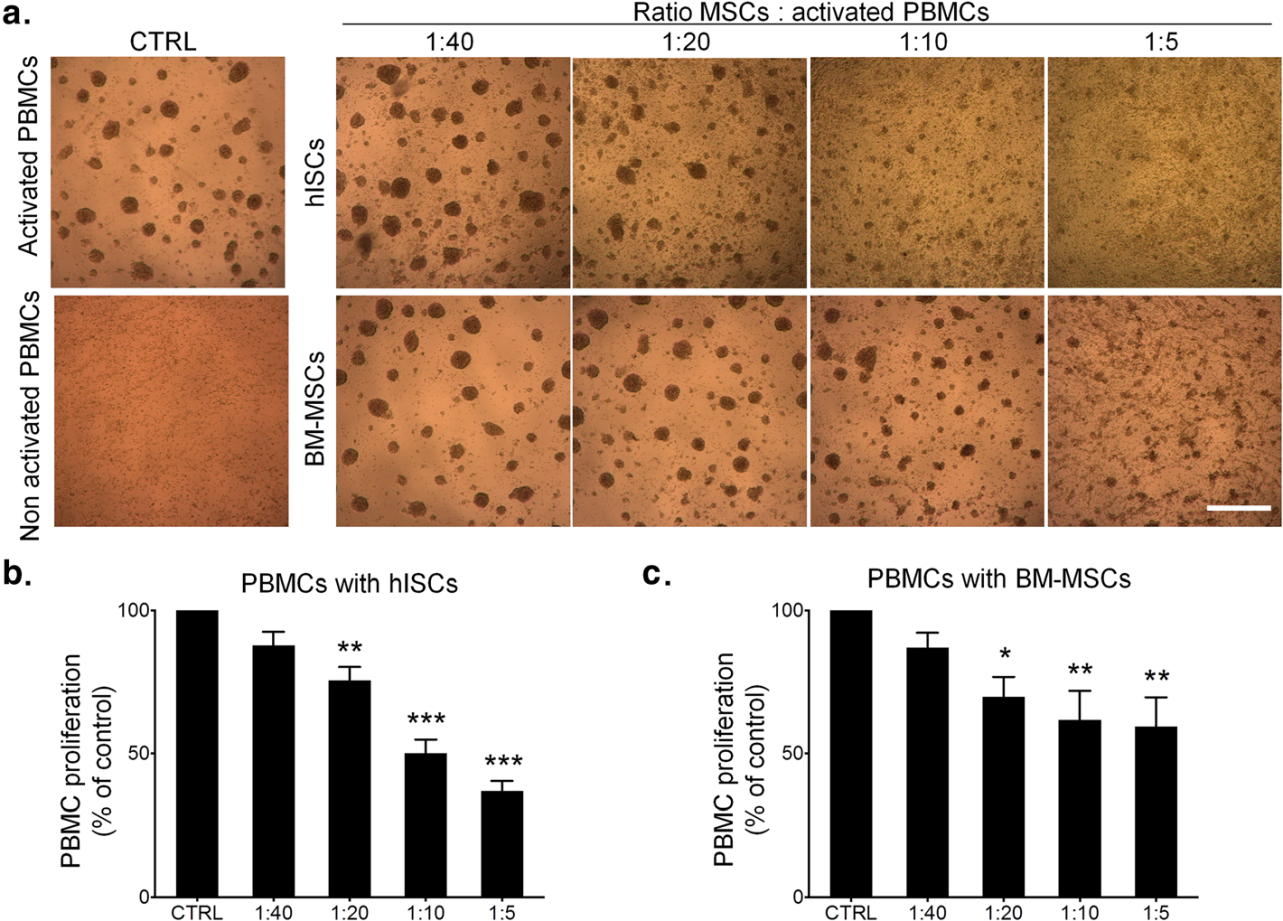
hISCs inhibit PBMCs proliferation. **a** Phase-contrast microscopic view of activated PBMCs after 96 h of co-culture with hISCs (top) or BM-MSCs (bottom). PHA activation induces the proliferation of PBMCs, which form suspended cell clusters (on the left). The number of PBMCs clusters decreases inversely with the ratio MSC to PBMC. The proliferation of PBMCs cultured alone (CTRL) or with hISCs or BM-MSCs at four ratios (1:5, 1:10, 1:20, 1:40; MSC to PBMC) was quantified. PBMCs proliferation was reduced by hISCs (**b**) and BM-MSCs (**c**) in co-culture. The inhibitory effect of both cell types was higher with increasing ratio. Results are representative of four independent experiments. *p < 0.05; **p < 0.001; ***p < 0.0001 when compared to the control. Scale bar = 500 μm

As hISCs share several phenotypic and some functional characteristics with MSCs, we aimed to analyze their immunomodulatory properties by looking at their ability to inhibit PBMCs proliferation. After 96 h co-culture of hISCs with PHA-activated PBMCs at different ratios, we measured the percentage of proliferative PBMCs. BM-MSCs were used as a positive control. PHA-activated PBMCs proliferated and formed clusters as depicted on Fig. 5a. The addition of hISCs or BM-MSCs led to a dose-dependent decrease of PBMC clusters (Fig. 5a). This dose-dependent inhibitory effect of hISCs or BM-MSCs on PBMCs was quantified by FACS. The highest 1:5 (MSC to PBMC) ratio reduced the proliferation rate of PBMCs by 63 ± 6.9% (*p* < 0.001, compared to control) and 40.6 ± 10.2% (*p* = 0.006) for hISCs and BM-MSCs respectively (Fig. 5b, c). MSCs can inhibit T cell proliferation or stimulate T-reg proliferation by secreting transforming growth factor-β (TGF-β), inducible indoleamine 2,3-dioxygenase (IDO), human leukocyte antigen class I molecule (HLA)-G5, prostaglandin E2 through cyclooxygenase (COX)-2, and TNFα-stimulated gene protein (TSG)-6, notably. Interestingly, RT-qPCR showed a comparable level of relative mRNA expression for these genes between MSCs and hISCs, arguing for a similar immunomodulatory profile (Supplementary Figure2).

### hISCs clusterize with MSCs on a heat map gene expression

**Fig. 6:**
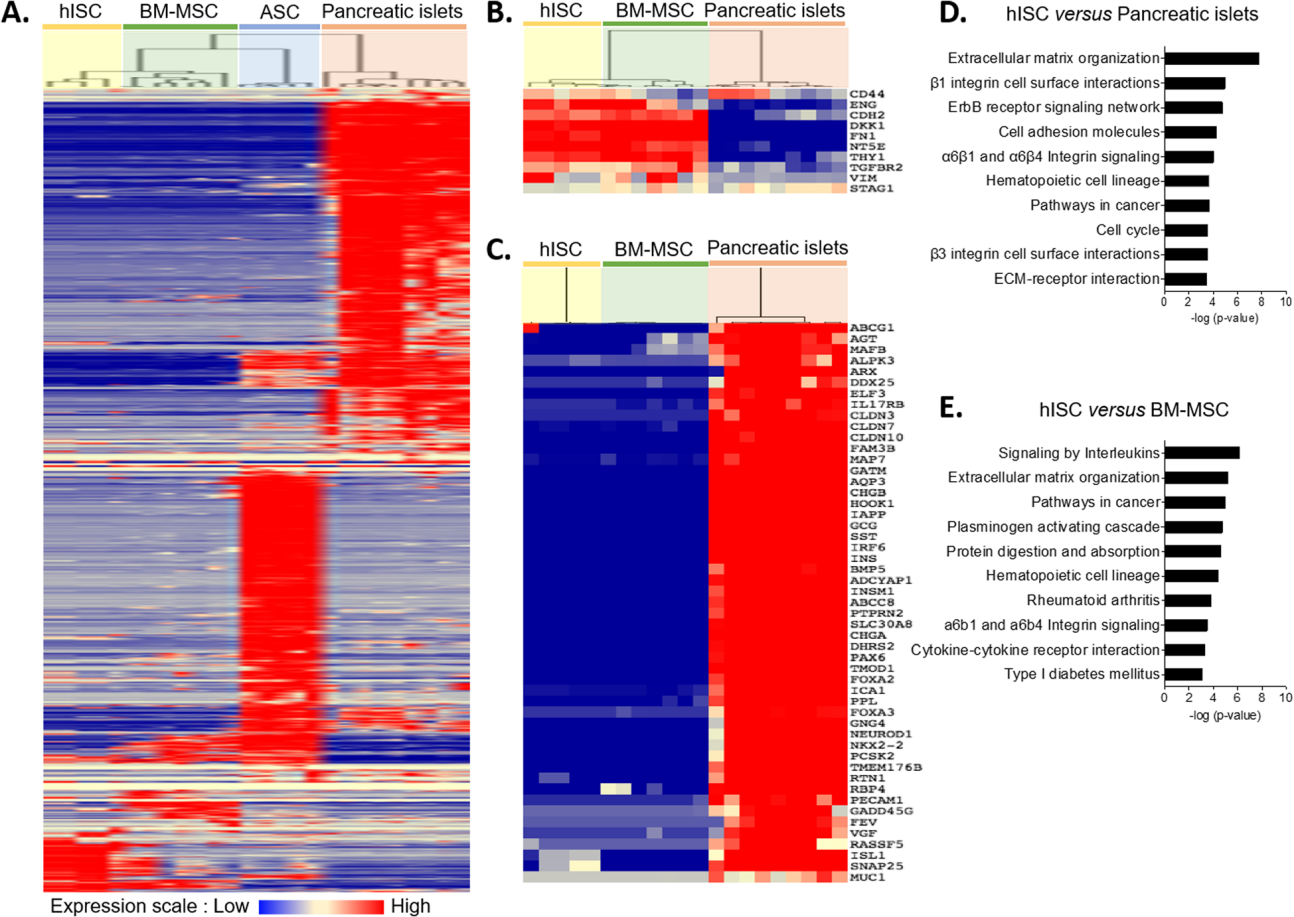
Hierarchical clustering shows a close profile of gene expression for hISCs and BM-MSCs. DNA chips from hISCs, BM-MSCs, ASCs, and pancreatic islets were used to perform hierarchical clustering. Normalized microarray data (a total of 4390 genes) were plotted as a heat map, with columns corresponding to individual arrays and rows depicting the level of probe set signal expression from low (deep blue) to high (deep red) gene expression. Clustering is indicated by the dendrogram on top: all 5 hISC subpopulations strongly cluster with BM-MSCs and not with pancreatic islet (**a**). Hierarchical clustering shows genes encoding for MSC markers (**b**) and endocrine markers (**c**), in hISCs, BM-MSCs, and pancreatic islets. Contrary to pancreatic islets, hISCs as well as BM-MSCs weakly express endocrine markers and strongly express genes related to MSC profile. Top 10 gene set enrichment analysis (ordered by *p* value) of overexpressed genes in hISCs compared to pancreatic islets (**d**) and BM-MSCs (**e**) were generated with the Reactome Functional Interaction Cytoscape plugin. High-expression gene category in hISCs includes the organization and interaction of the ECM

Taking into account the incomplete ability of hISCs to differentiate into the three lineages, a comparative transcriptomic analysis with other sources of MSCs such as BM-MSCs and ASCs and with pancreatic islets was performed to deepen our observations on the potential mesenchymal profile of hISCs. A total of 4360 genes differentially expressed were retained for the analysis. The hierarchical clustering analysis delineated two major clusters: one cluster comprising hISCs, BM-MSCs, and ASCs, and another one comprising pancreatic islets. The hISCs strongly clusterized with BM-MSCs and slightly less with ASCs (Fig. 6a). The clustering approach was validated by introducing, into this first analysis, public DNA chips from human pancreatic islet cells after in vitro expansion (EXP islets) produced by three different expansion protocols [22]. Hierarchical analysis confirmed that hISCs, BM-MSCs, and EXP islets clusterized together and not with pancreatic islets. Gene expression of hISCs was also very close to EXP islet from Bodnar et al. [33] expansion protocol (Supplementary Figure3). No engagement of hISCs towards endocrine or pancreas differentiation was observed at the mRNA level (Supplementary Figure 4).

In addition, the transcriptomic analysis confirmed the expression of the specific MSC markers CD73 (Ecto-5-prime-nucleotidase, NT5E), CD90 (thymocyte differentiation antigen 1, THY 1), CD105 (endoglin, ENG), and vimentin (VIM) (Fig. 6b). Finally, we observed a very low level of endocrine genes expression in hISCs as compared to pancreatic islets (Fig. 6c).

Using gene expression microarrays and SAM analysis, we found 450 and 337 genes that were significantly overexpressed in hISCs as compared to pancreatic islets and BM-MSCs, respectively. Finally, biological pathways analysis from transcriptomic data (Reactome analysis) revealed a significant enrichment of genes involved in the organization and interaction of the ECM (Fig. 6d, e). Pathway enrichment analyses are detailed in Supplementary Tables 1–4. Among overexpressed genes in hISCs, we highlighted those that code for type I, IV, and VI collagen isoforms, as well as MMP-9. All these proteins are present in human pancreas ECM.

### hISCs express and secrete ECM proteins

**Fig. 7:**
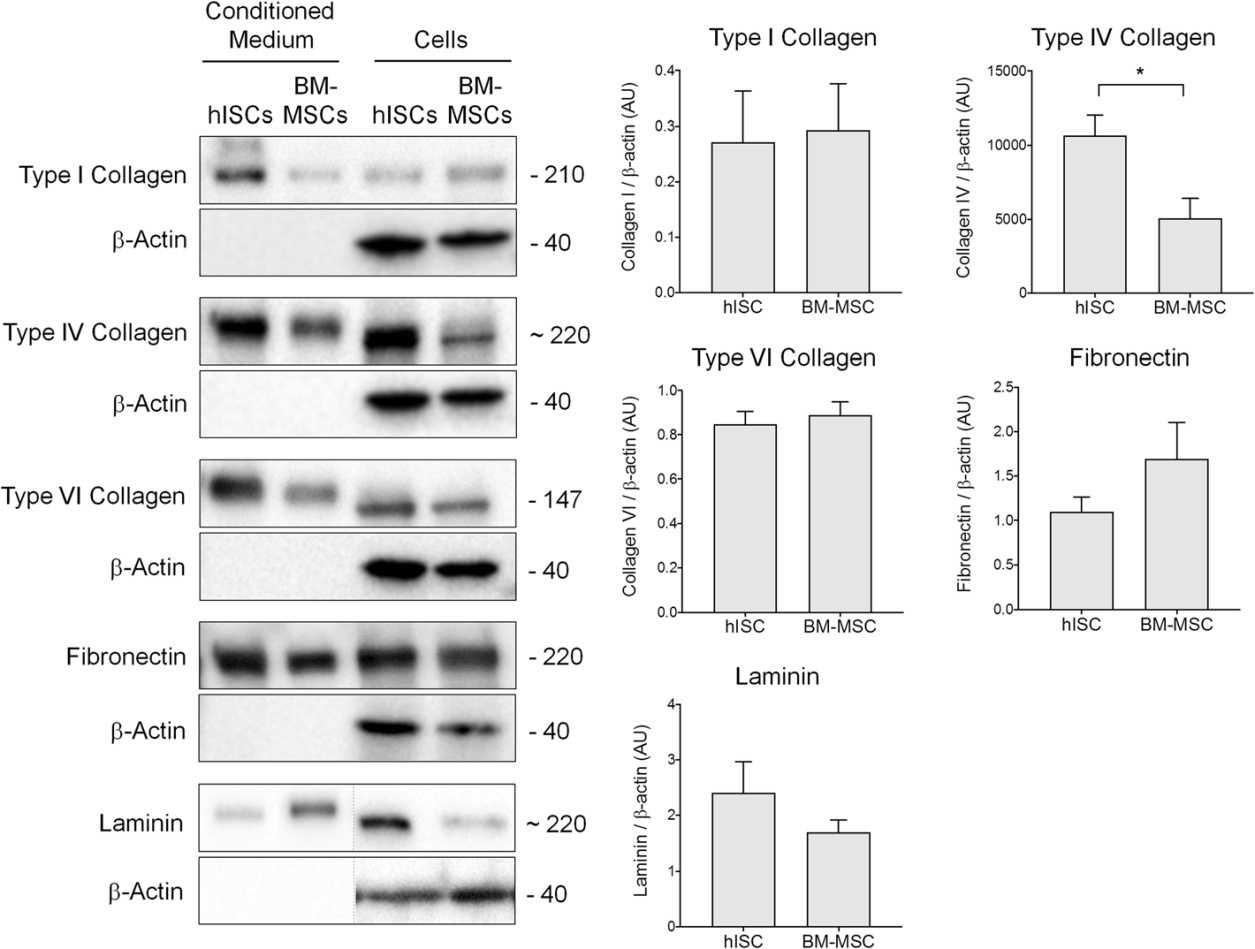
hISCs secrete pancreatic ECM proteins. Extracted proteins from hISCs and BM-MSCs and corresponding conditioned media were analyzed by western blotting. Type I, IV, and VI collagens as well as fibronectin and laminin were detected in both cell types but at different levels of expression. β-actin was used as loading control. The right panels represent the quantification of the Western blot, *n* = 4, *p < 0.05, significant differences

It has been suggested that MSCs may influence cell behavior in part by secreting specific ECM proteins [10]. To confirm the previous transcriptomic analysis, we investigated the ability of hISCs to secrete ECM proteins identified at the transcriptomic level. To this aim, protein extracts and conditioned media from hISCs were collected and analyzed by western blotting (Fig. 7). Three different collagen isoforms were detected with a single band at 210 kDa, 220 kDa, and 147 kDa for type I, IV, and VI collagens, respectively. Fibronectin and laminin were also expressed and secreted by hISCs, with a single band at 220 kDa. All of these proteins were also expressed and secreted by BM-MSCs. However, the expression level of type IV collagen was significantly twice as high in hISCs as in BM-MSCs (*p* = 0.04). Interestingly, some of the secreted proteins showed a slight shift in molecular weight relative to the proteins extracted from the corresponding cells. In these experiments, only traces of actin were detected in the conditioned medium, excluding the possibility of cellular contamination.

## Discussion

In this study, we characterized immortalized human islet-derived stromal cells (hISCs) that exhibited a fibroblast-like shape with high adhesive properties and virtually unlimited proliferative potential. We demonstrated that hISCs shared phenotypic markers and immunomodulatory properties with BM-MSCs, while being distinct in their differentiation potential, and ECM protein expression signature. Thus, hISCs seem to represent an endless source of human cells showing some MSC characteristics while retaining a pancreatic identity.

Accumulating evidence suggested the presence of proliferative cells exhibiting a mesenchymal phenotype after several days of culture of highly pure adult human islets [21, 22]. Previous studies described these cells as a new source of insulin-producing cells by re-differentiation in a mesenchymal-epithelial transition way [34–36]. As the re-differentiation protocols were carried out with more or less success in the literature, these cells are now considered mainly as supporting cells with interesting secretory properties. We hypothesized that these cells may be MSCs that are known to be present in virtually all tissues and may play an important role in maintaining tissue homeostasis [37]. According to the original studies of Friedenstein and Pittenger [38, 39], we used their physical property of plastic adherence to isolate adherent cells from human pancreatic islet. To overcome the limited access of human pancreatic islets and the early senescence highlighted in pancreatic human MSCs after ten passages [40, 41], we immortalized islet-derived cells by using hTERT-expressing retrovirus.

Phenotypic profile of hISCs meets the minimum criteria defining MSCs [1] with adhesion to plastic and expression of a characteristic surface antigen pattern. Using standard differentiation protocols, we succeeded to induce hISCs towards osteogenesis and adipogenesis, at least to a committed intermediate stage of differentiation. It is noteworthy that adipocyte differentiation ability can disappear in MSC after several passages. Indeed, Seeberger and colleagues observed a loss in cellular plasticity, with an inability of the pancreatic MSCs to differentiate into adipocytes after 4 passages [42]. As our differentiation protocols were applied to hISCs at passages 15–20, this could explain why we were not able to induce complete adipogenesis. Moreover, Rauch and colleagues demonstrated that adipogenesis within MSC is notably restricted, probably because it requires considerably more dramatic transcriptional changes than osteogenesis in MSCs [43]. In addition, we cannot exclude that hTERT transgene affects the cellular plasticity of hISCs. Finally, differentiation properties of MSCs may be also impacted by the donor characteristics [44] and methods used for isolation, culture, and expansion of cells [16]. This heterogeneity was also demonstrated in stromal cells expanded from adult human islets maintained in culture [45]. In this population, Russ found that, on average, only 5.5% of these cells at different passages could be classified as true MSCs, as judged by their ability to differentiate into adipocytes or osteoblasts. More generally, MSCs may have an inherent differentiation potential according to their tissue origin [46]. For instance, BM-MSCs showed a greater capacity to generate bone and weaker ability to form lipid droplets as compared to ASCs [47]. This observation is in accordance with the closer clustering of hISCs with BM-MSCs than the ASCs in our transcriptomic analysis.

Hence, the unsupervised hierarchical clustering obtained by hybridization of microarrays from hISCs, ASCs, BM-MSCs and human pancreatic islets revealed that gene expression profiles are clearly separated. Importantly, hISCs are closer to MSCs transcriptomic profiles than to their native pancreatic tissue origin. All MSC-like cells seem to share core gene markers but keep a differential gene expression profiles in a tissue-related manner. In supplementary analysis, we tested clustering with public microarray from three different protocols of expanded human pancreatic islets cells in monolayer (EXP) available from Kutlu’s meta-analysis [22]. Interestingly, we demonstrate considerable overlap between the hISCs and EXP cells of the Bodnar protocol [33] although the culture medium used to expand the cells from whole islets is different from ours. In accordance with this meta-analysis, we demonstrated that hISCs express genes that specifically code for proteins involved in structural ECM organization, cell adhesion, and ECM-derived differentiation pathways. Conversely, most of the genes encoding the hormones or proteins involved in their secretion—specificity of islet expression—were not expressed in hISCs. In conclusion, according to the strict defined criteria for MSCs, hISCs should be considered MSC-like rather than true MSCs.

If hISCs and BM-MSCs are close according to their transcriptomic profile, genes involved in ECM organization were nevertheless significantly different. In this sense, a comparative and quantitative proteomic analysis demonstrated that ECM produced in vitro by BM-MSCs, ASCs, or fibroblasts shared a common set of proteins but also displayed a specific and unique matrisome signature depending on their tissue origin [48]. Among the genes most strongly expressed in hISCs, we found those encoding for type IV and VI collagen isoforms. In addition to these two proteins, WB analysis of hISC-conditioned medium identified other ECM proteins including type I collagen, fibronectin, and laminin. Interestingly, all of these proteins are normally expressed in abundance in the pancreas, around and within the islets [49, 50]. This observation supports the fact that hISCs retained some “pancreatic memory” at least for ECM proteins. This property highlights hISCs as very interesting supportive cells that can potentially reconstitute a pancreatic microenvironment close to that of origin. They can therefore be a useful tool for the maintenance of islets in culture as well as for the conditioning of islets intended to be grafted. Previous studies have shown the beneficial contribution of ECM proteins for human islet biology. Enrichment of encapsulated human pancreatic islets cells with type VI collagen improved islet survival and function [51]. Type I/IV collagens and fibronectin induced the adhesion of human islets, and insulin release was higher on fibronectin coating [52]. Although synthetic or purified individual ECM proteins have undeniable effects on the islets, they are probably limited and insufficient alone to fully replicate the original ECM environment [53]. Indeed, at least 120 proteins were identified within the human pancreatic matrisome [54]. Moreover, in addition to its adhesive and supportive structure, the native ECM serves also as a “reservoir” of biologically sequestered active molecules, such as cytokines and growth factors [55]. For example, an experimental study has recently shown that ANXA-1 protein trapped in the ECM can promote some of  the beneficial effects of ASC-conditioned medium on human islet function [10].

Finally, in the context of islet transplantation, hISCs could improve islet engraftment thanks to their immunomodulatory properties. Immunosuppressive effects of MSCs act mainly through paracrine mechanism and depend on the secretion of soluble factors such as prostaglandin E2, interleukin (IL)-6, IL-1Ra, TGF-β1, tumor necrosis factor-inducible gene 6 protein, and HGF [56, 57]. Indeed, it has been reported previously that human pancreatic MSCs can protect islets from pro-inflammatory cytokines with the putative effect of secreted cytoprotective factors including HGF, IL-6, MMP-1, MMP-2, and MMP-9, in vitro [9]. We confirmed in part these observations in hISCs, but further analysis of the secretome of hISCs should help to clarify their potential interest in islet transplantation.

## Conclusion

In conclusion, although the BM-MSCs have demonstrated their potential interest in diabetes cell therapy, we believe that the new hISC cell line derived from the pancreas and more precisely from human islets represent a major asset. Thanks to their “pancreatic memory,” they can participate in the reconstitution of a microenvironment very close to the one observed within the pancreas. This represents a real benefit for a potential application, in a cell-free strategy, in the context of islet transplantation. The development of therapeutic strategies that may use cell-free secreted products will indeed attenuate the safety concerns relative to the use of living stem cells, in cell therapy applications.

## Supplementary information

**Additional file 1: Supplementary Figure 1.** Map of the pEGN-hTERT vector containing a fusion gene coding for EGFP and neomycin resistance.

**Additional file 2: Supplementary Figure 2.** Relative expression of immunomodulatory genes in hISCs and BM-MSCs. RT-qPCR for transforming growth factor-β (TGF-β), hepatocyte growth factor (HGF), inducible indoleamine 2,3-dioxygenase (IDO), human leukocyte antigen class I molecule (HLA)-G5, Cyclo oxygenase COX-2 (promoting prostaglandin PG E2 formation), interleukin (IL)-6 and TNFα-stimulated gene protein (TSG)-6 showed a comparable level of relative mRNA expression in MSCs and hISCs.

**Additional file 3: Supplementary Figure 3.** Hierarchical clustering demonstrates a great commonality between hISCs, EXP-islets and BM-MSCs. Data from DNA chips from hISCs, BM-MSCs, ASCs, human pancreatic islets and pancreatic islet after in vitro expansion (EXP islets, in silico *data*) were used to perform hierarchical clustering. All 5 hISC populations strongly cluster with EXP islets and BM-MSCs.

**Additional file 4: Supplementary Figure 4.** Expression of endocrine lineage genes in hISCs, BM-MSCs and human islets. RT-qPCR for insulin, glucagon and chromogranin A did not show endocrine differentiation in hISCs compared to human islets. Expression of factors of transcription involved in endocrine differentiation (PAX6, ISL-1, NEUROG 3, NeuroD1, MAFA, PTF-1, Nkx6–1, Nkx2–2) were comparable between hISCs and BM-MSCs.

**Additional file 5: Supplementary Table 1.** Top 5 gene sets significantly enriched in hISCs compared to human pancreatic islets. Using the significance analysis of microarrays (SAM) software, 450 genes were significantly overexpressed in hISC versus human islets. Gene annotation and networks (ordered by *P*-value) were generated with the Reactome Functional Interaction Cytoscape plugin. **Supplementary Table 2.** Top 5 gene sets significantly enriched in human pancreatic islets compared to hISCs. Using the significance analysis of microarrays analysis (SAM) software, 1580 genes were significantly overexpressed in hISCs versus human islets. Gene annotation and networks (ordered by *P*-value) were generated with the Reactome Functional Interaction Cytoscape plugin. **Supplementary Table 3**. Top 5 gene sets significantly enriched in hISCs compared to BM-MSCs. Using the significance analysis of microarrays (SAM) software, 337 genes were significantly overexpressed in hISCs versus human islets. Gene annotation and networks (ordered by *P*-value) were generated with the Reactome Functional Interaction Cytoscape plugin. **Supplementary Table 4.** Top 5 gene sets significantly enriched in BM-MSCs compared to hISCs. Using the significance analysis of microarrays (SAM) software, 276 genes were significantly overexpressed in hISCs versus human islets. Gene annotation and networks (ordered by *P*-value) were generated with the Reactome Functional Interaction Cytoscape plugin. **Supplementary Table 1.** Top 5 gene sets significantly enriched in hISCs compared to human islets.

## Data Availability

The majority of the data generated or analyzed during this study are included in this article. Unpublished data are available from the corresponding author upon reasonable request.

## Abbreviations

APC: Allophycocyanin
ASC: Adipose stromal cells
AU: Arbitrary unit
BM-MSC: Bone marrow mesenchymal stromal cells
ECM: Extracellular matrix
EGF: Epidermal growth factor
FABP4: Fatty acid-binding protein 4
FDR: False discovery rate
FGF: Fibroblast growth factor
GEO: Gene expression omnibus
GFP: Green fluorescent protein
hISC: Human islet-derived stromal cells
hTERT: Human telomerase reverse transcriptase
IL: Interleukin
LPL: Lipoprotein lipase
MMP: Matrix metalloproteinases
MSC: Mesenchymal stromal cells
PE: Phycoerythrin
PerCP: Peridinin chlorophyll protein complex
PHA: Phytohemagglutinin
PBMC: Peripheral blood mononuclear cells
PPARγ: Peroxisome proliferator-activated receptor gamma
SAM: Significance Analysis of Microarrays
SEM: Standard error of the mean
TGF: Transforming growth factor
