# Correction to: Characterization of immortalized human islet stromal cells reveals a MSC-like profile with pancreatic features

**DOI:** 10.1186/s13287-020-01717-4

**Published:** 2020-05-21

**Authors:** Orianne Villard, Mathieu Armanet, Guilhem Couderc, Claire Bony, Jerome Moreaux, Daniele Noël, John De Vos, Bernard Klein, Jean-Luc Veyrune, Anne Wojtusciszyn

**Affiliations:** 1 Laboratory of Cell Therapy for Diabetes, Institute of Regenerative Medicine and Biotherapy, Univ. Montpellier, CHU Montpellier, Montpellier, France; 2 Department of Endocrinology, Diabetes, and Nutrition, Univ. Montpellier, CHU Montpellier, Montpellier, France; 3 Cell Therapy Unit, Hospital Saint- Louis, AP-HP, Paris, France; 4 Department of Endocrinology, Diabetology and Metabolism, Lausanne University Hospital, 8 avenue de la Sallaz -, 1011 Lausanne, Switzerland; 5 Department of Biological Haematology, Univ. Montpellier, CHU Montpellier, Montpellier, France; 6 Department of Cell and Tissue Engineering, Univ. Montpellier, CHU Montpellier, Montpellier, France; 7 IRMB, INSERM U 1183, Univ Montpellier, INSERM, Montpellier, France; 8 IGH, Univ Montpellier, CNRS, Montpellier, France; 9 Laboratory of Cell Therapy for Diabetes, Institute of Regenerative Medicine and Biotherapy, Univ. Montpellier, CHU Montpellier, Montpellier, France; 10 Department of Endocrinology, Diabetes, and Nutrition, Univ. Montpellier, CHU Montpellier, Montpellier, France; 11 Department of Endocrinology, Diabetology and Metabolism, Lausanne University Hospital, 8 avenue de la Sallaz, 1011 Lausanne, Switzerland

**Correction to: Stem Cell Res Ther (2020)11: 158**

**https://doi.org/10.1186/s13287-020-01649-z**

The original article [1] presents co-author John De Vos’s name incorrectly. The correct presentation (with a space between ‘De’ and ‘Vos’) can be seen in this correction article.
