# Supplementary figures and images for: Characterization of immortalized human islet stromal cells reveals a MSC-like profile with pancreatic features
Source: Stem Cell Res Ther. 2020 Apr 17;11:158. doi: 10.1186/s13287-020-01649-z (PMC7165390; doi:10.1186/s13287-020-01649-z)

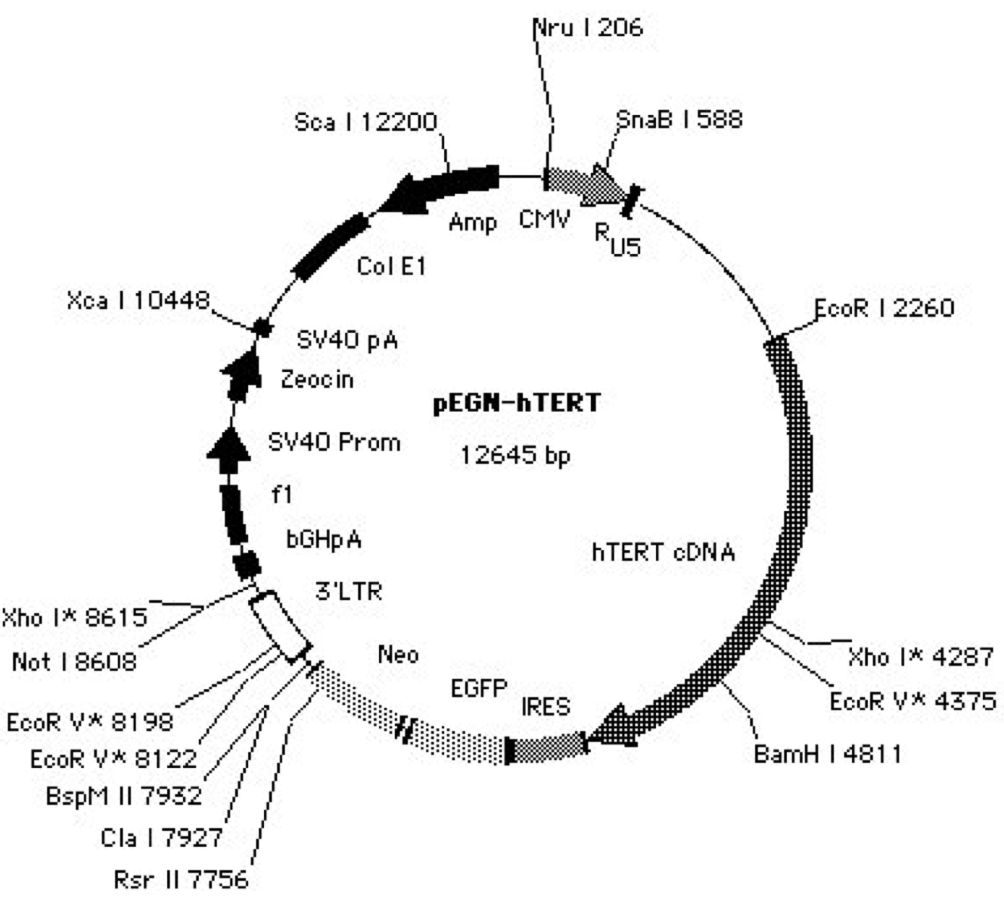

Supplement: Supplementary file 1 — Additional file 1: Supplementary Figure 1. Map of the pEGN-hTERT vector containing a fusion gene coding for EGFP and neomycin resistance. [file 13287_2020_1649_MOESM1_ESM.tif]

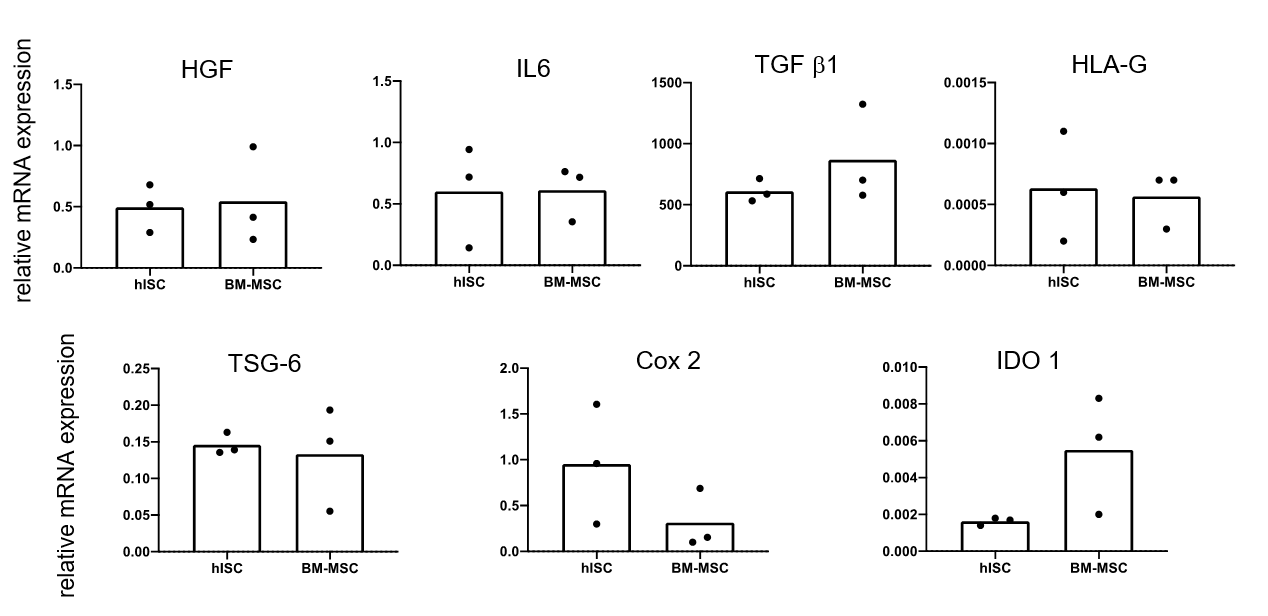

Supplement: Supplementary file 2 — Additional file 2: Supplementary Figure 2. Relative expression of immunomodulatory genes in hISCs and BM-MSCs. RT-qPCR for transforming growth factor-β (TGF-β), hepatocyte growth factor (HGF), inducible indoleamine 2,3-dioxygenase (IDO), human leukocyte antigen class I molecule (HLA)-G5, Cyclo oxygenase COX-2 (promoting prostaglandin PG E2 formation), interleukin (IL)-6 and TNFα-stimulated gene protein (TSG)-6 showed a comparable level of relative mRNA expression in MSCs and hISCs. [file 13287_2020_1649_MOESM2_ESM.tif]

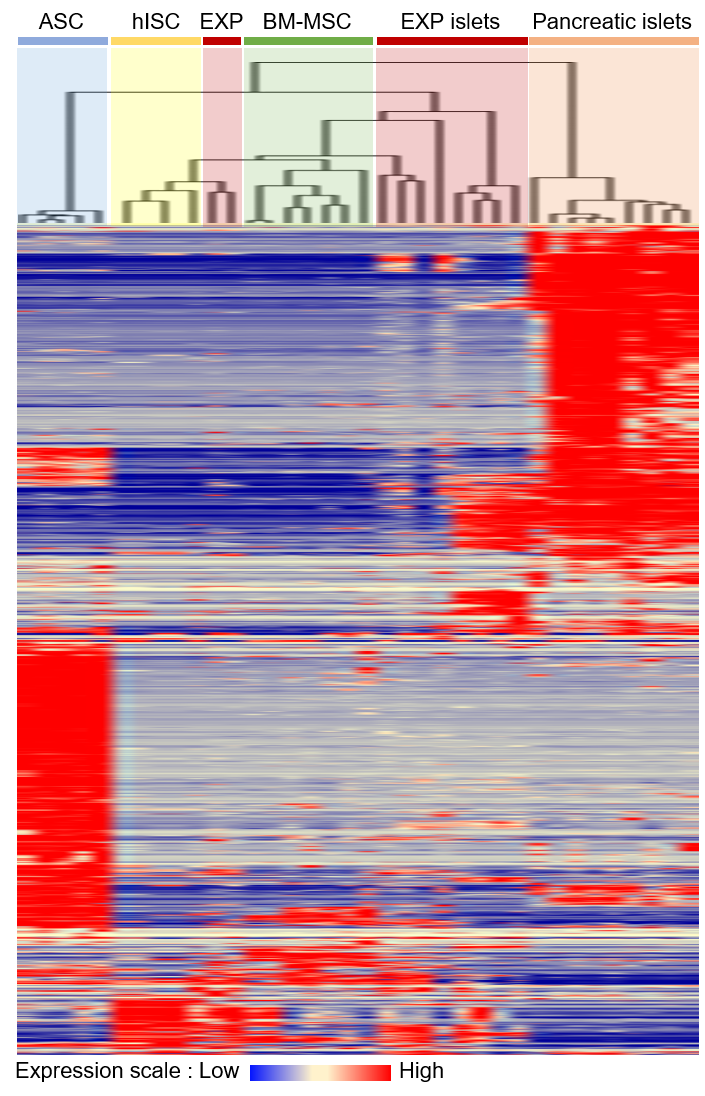

Supplement: Supplementary file 3 — Additional file 3: Supplementary Figure 3. Hierarchical clustering demonstrates a great commonality between hISCs, EXP-islets and BM-MSCs. Data from DNA chips from hISCs, BM-MSCs, ASCs, human pancreatic islets and pancreatic islet after in vitro expansion (EXP islets, in silico data) were used to perform hierarchical clustering. All 5 hISC populations strongly cluster with EXP islets and BM-MSCs. [file 13287_2020_1649_MOESM3_ESM.tif]

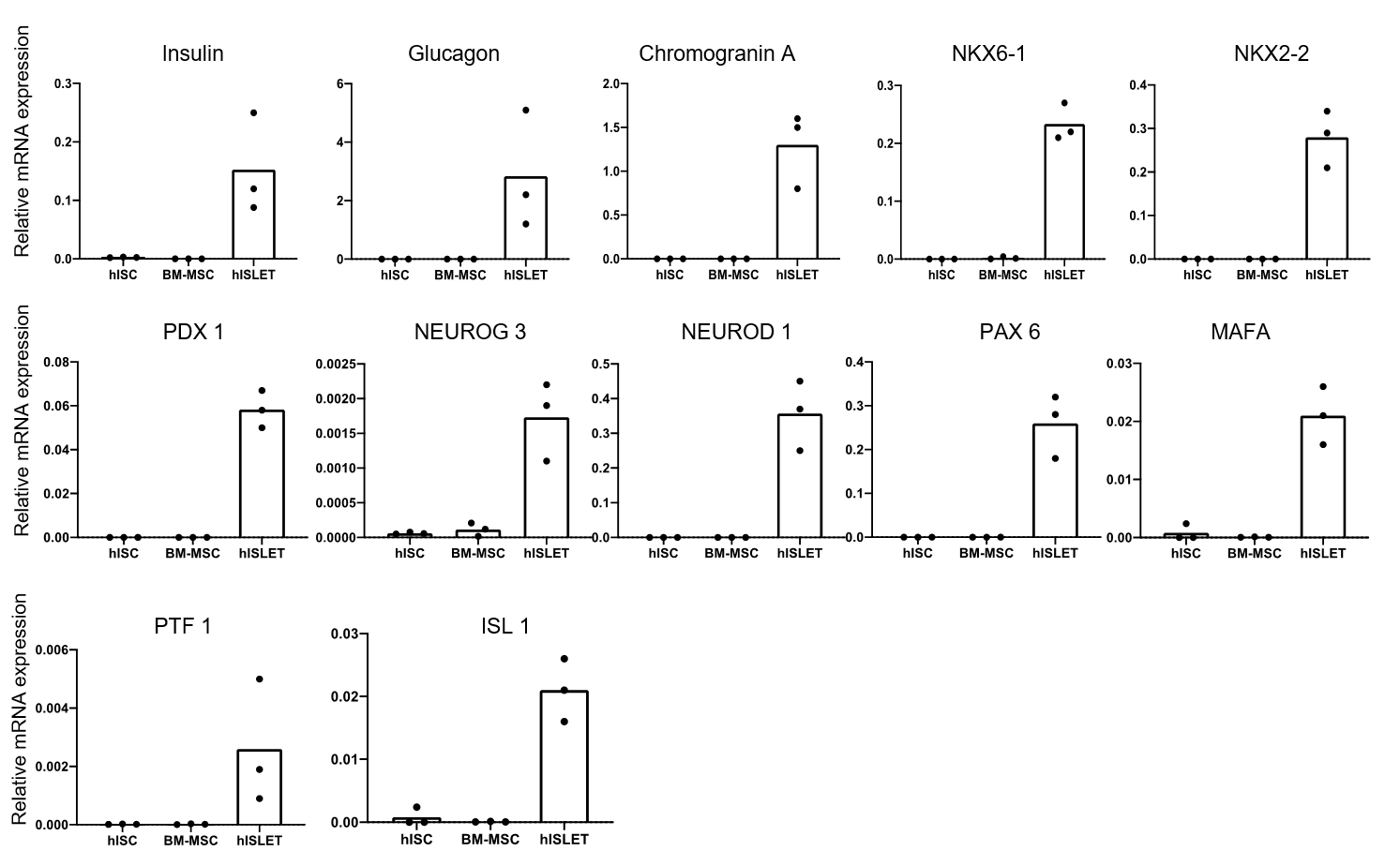

Supplement: Supplementary file 4 — Additional file 4: Supplementary Figure 4. Expression of endocrine lineage genes in hISCs, BM-MSCs and human islets. RT-qPCR for insulin, glucagon and chromogranin A did not show endocrine differentiation in hISCs compared to human islets. Expression of factors of transcription involved in endocrine differentiation (PAX6, ISL-1, NEUROG 3, NeuroD1, MAFA, PTF-1, Nkx6–1, Nkx2–2) were comparable between hISCs and BM-MSCs. [file 13287_2020_1649_MOESM4_ESM.tif]
