# Supplementary material for: Characterization of immortalized human islet stromal cells reveals a MSC-like profile with pancreatic features
Source: Stem Cell Res Ther. 2020 Apr 17;11:158. doi: 10.1186/s13287-020-01649-z (PMC7165390; doi:10.1186/s13287-020-01649-z)
Supplement: Supplementary file 5 — Additional file 5: Supplementary Table 1. Top 5 gene sets significantly enriched in hISCs compared to human pancreatic islets. Using the significance analysis of microarrays (SAM) software, 450 genes were significantly overexpressed in hISC versus human islets. Gene annotation and networks (ordered by P-value) were generated with the Reactome Functional Interaction Cytoscape plugin. Supplementary Table 2. Top 5 gene sets significantly enriched in human pancreatic islets compared to hISCs. Using the significance analysis of microarrays analysis (SAM) software, 1580 genes were significantly overexpressed in hISCs versus human islets. Gene annotation and networks (ordered by P-value) were generated with the Reactome Functional Interaction Cytoscape plugin. Supplementary Table 3. Top 5 gene sets significantly enriched in hISCs compared to BM-MSCs. Using the significance analysis of microarrays (SAM) software, 337 genes were significantly overexpressed in hISCs versus human islets. Gene annotation and networks (ordered by P-value) were generated with the Reactome Functional Interaction Cytoscape plugin. Supplementary Table 4. Top 5 gene sets significantly enriched in BM-MSCs compared to hISCs. Using the significance analysis of microarrays (SAM) software, 276 genes were significantly overexpressed in hISCs versus human islets. Gene annotation and networks (ordered by P-value) were generated with the Reactome Functional Interaction Cytoscape plugin. Supplementary Table 1. Top 5 gene sets significantly enriched in hISCs compared to human islets. [file 13287_2020_1649_MOESM5_ESM.docx]

**Supplementary Table 1 Top 5 gene sets significantly enriched in hISCs compared to human islets**

| Gene set name | # Genes in Gene Set (K) | # Genes in Overlap (k) | k/K | Genes | *P*-value | FDR  *q*-value |
| --- | --- | --- | --- | --- | --- | --- |
| Extracellular matrix organization | 255 | 22 | 0.0235 | PRSS2, CTRB2, COL1A2, COL10A1, BGN, CEACAM6, DDR2, JAM3, COL22A1, CDH1, LOX, COL4A5, TGFB2, ITGA11, HAPLN1, FGA, ITGA4, COL6A1, ITGA6, PLEC, THBS1, TTR | 1.7 E-08 | 9.5 E-06 |
| β1 integrin cell surface interactions | 66 | 9 | 0.0061 | COL1A2, COL4A5, ITGA11, CSPG4, FGA, ITGA4, COL6A1, ITGA6, THBS1 | 1.0 E-05 | 2.9 E-03 |
| ErbB receptor signaling network | 15 | 5 | 0.0014 | EREG, TGFA, EGFR, NRG1, ERBB3 | 1.1 E-05 | 3.1 E-03 |
| Cell adhesion molecules (CAMs) | 145 | 12 | 0.0133 | CLDN1, CLDN7, L1CAM, OCLN, PDCD1LG2, JAM3, CDH1, VTCN1, ITGA4, ITGA6, NRXN3, CD4 | 4.9 E-05 | 7.0 E-03 |
| β6α1 and β6α4 Integrin signaling | 35 | 6 | 0.0032 | SFN, IL1A, EGFR, CDH1, ITGA6, ERBB3 | 9.1 E-05 | 0.0105 |

**Supplementary Table 2. Top 5 gene sets significantly enriched in human islets compared to hISCs**

| Gene set name | # Genes in Gene Set (K) | # Genes in Overlap (k) | k/K | Genes | *P*-value | FDR  *q*-value |
| --- | --- | --- | --- | --- | --- | --- |
| Pancreatic secretion | 96 | 26 | 0.0088 | CELA3A, CELA3B, FXYD2, CPA2, CPA1, CPB1, CTRB1, KCNQ1, CTRL, GNAS, PNLIPRP1, PNLIPRP2, ATP2A3, CA2, CELA2A, CELA2B, PLA2G1B, PRSS1, PRSS2, CEL, PLCB4, ADCY2, ADCY1, CHRM3, CFTR, PNLIP | 4.3 E-11 | 9.5 E-06 |
| Maturity onset diabetes of the young | 26 | 14 | 0.0024 | MNX1, RFX6, HHEX, HNF4G, HNF4A, NR5A2, GCK, FOXA3, FOXA2, INS, NKX2-2, PAX6, NEUROD1, SLC2A2 | 3.0 E-10 | 2.9 E-03 |
| Insulin secretion | 85 | 21 | 0.0078 | KCNMB2, FXYD2, RAPGEF4, ADCYAP1, SNAP25, PCLO, GCG, GCK, CACNA1D, GNAS, CREB5, INS, PLCB4, ADCY2, ADCY1, CHRM3, RIMS2, FFAR1, SLC2A2, ABCC8, CAMK2B | 1.5 E-08 | 3.1 E-03 |
| Regulation of β-cell development | 31 | 13 | 0.0029 | RFX6, HNF4G, HNF4A, NR5A2, GCK, FOXA3, FOXA2, INS, NKX2-2, PAX6, NEUROD1, INSM1, SLC2A2 | 2.4 E-08 | 7.0 E-03 |
| FOXA2 and FOXA3 transcription factor networks | 40 | 12 | 0.0037 | PCK1, HNF4A, GCK, FOXA3, FOXA2, INS, ALB, TTR, ALDOB, SLC2A2, ABCC8, DLK1 | 2.7 E-07 | 0.0105 |

**Supplementary Table 3. Top 5 gene sets significantly enriched in** **hISCs compared to BM-MSCs**

| Gene set name | # Genes in Gene Set (K) | # Genes in Overlap (k) | k/K | Genes | *P*-value | FDR  *q*-value |
| --- | --- | --- | --- | --- | --- | --- |
| Signaling by Interleukins | 460 | 24 | 0.042 | EREG, IL24, NEFL, IL11, IL1A, IL1B, PTAFR, PTGS2, EGFR, IL33, CSF2, FGF5, HGF, RASGRP1, IL7R, MMP1, MMP3, IRAK3, TNFRSF1B, MMP9, BIRC5, CBL, ERBB3, CD4 | 6.2 E-07 | 3.3 E-04 |
| Extracellular matrix organization | 255 | 16 | 0.024 | PRSS2, CTSS, CEACAM6, COL22A1, COL4A6, COL4A5, BMP2, COL5A3, FGA, MMP1, MMP3, MMP9, COL6A1, PECAM1, ITGA6, COL15A1 | 5.6 E-06 | 1.5 E-03 |
| Pathways in cancer | 397 | 20 | 0.037 | EDNRA, EDNRB, AR, AGTR1, PTGER3, PTGS2, EGFR, FGF5, HGF, COL4A6, COL4A5, RASGRP1, BMP2, MMP1, MMP9, BIRC5, ITGA6, LEF1, CBL, GNG4 | 9.6 E-06 | 1.7 E-03 |
| Plasminogen activating cascade | 9 | 4 | 0.001 | SERPINB2, FGA, MMP1, MMP3 | 1.7 E-05 | 2.2 E-03 |
| Protein digestion and absorption | 90 | 9 | 0.008 | PRSS2, COL22A1, SLC8A1, CPA2, COL4A6, COL4A5, COL5A3, COL6A1, COL15A1 | 2.1 E-05 | 2.2 E-03 |

**Supplementary Table 4. Top 5 gene sets significantly enriched in BM-MSCs compared to hISCs**

| Gene set name | # Genes in Gene Set (K) | # Genes in Overlap (k) | k/K | Genes | *P*-value | FDR  *q*-value |
| --- | --- | --- | --- | --- | --- | --- |
| Extracellular matrix organization | 96 | 26 | 0.0088 | COL12A1, COL8A2, COL8A1, FN1, ELN, COL21A1, DDR2, JAM2, COMP, EFEMP1, LOX, FBLN1, FBLN2, TGFB2, COL5A1, ITGA11, COL14A1, HAPLN1, ADAMTS5, COL6A1, THBS1, ACAN, MFAP5, MFAP4 | 2.2 E-16 | 8.2 E-14 |
| Integrin signalling pathway | 26 | 14 | 0.0024 | COL12A1, COL8A2, COL8A1, FN1, ITGBL1, COL5A1, ITGA11, COL14A1, COL6A1 | 4.5 E-05 | 5.4 E-03 |
| Protein digestion and absorption | 85 | 21 | 0.0078 | COL12A1, ELN, SLC7A8, COL21A1, COL5A1, COL14A1, COL6A1 | 4.8 E-05 | 5.4 E-03 |
| Proteoglycans in cancer | 31 | 13 | 0.0029 | PLCE1, ANK2, FN1, IGF1, COL21A1, TLR4, TGFB2, HSPB2, MET, THBS1 | 6.1 E-05 | 5.4 E-03 |
| β1 integrin cell surface interactions | 40 | 12 | 0.0037 | FN1, JAM2, COL5A1, ITGA11, COL6A1, THBS1 | 7.3 E-05 | 5.4 E-03 |
